# *Cis*–*trans* isomerism modulates the magnetic relaxation of dysprosium single-molecule magnets†
†Electronic supplementary information (ESI) available: Experimental details and additional figures (Tables S1–S6 and Fig. S1–S25). CCDC 1041137–1041139. For ESI and crystallographic data in CIF or other electronic format see DOI: 10.1039/c5sc04510j


**DOI:** 10.1039/c5sc04510j

**Published:** 2016-02-16

**Authors:** Jianfeng Wu, Julie Jung, Peng Zhang, Haixia Zhang, Jinkui Tang, Boris Le Guennic

**Affiliations:** a State Key Laboratory of Rare Earth Resource Utilization , Changchun Institute of Applied Chemistry , Chinese Academy of Sciences , Changchun 130022 , P. R. China . Email: tang@ciac.ac.cn; b Institut des Sciences Chimiques de Rennes , UMR 6226 CNRS-Université de Rennes 1 , 263 Avenue du General Leclerc , 35042 Rennes Cedex , France . Email: boris.leguennic@univ-rennes1.fr; c University of Chinese Academy of Sciences , Beijing , 100049 , P. R. China

## Abstract

Geometry and magnetic relaxation modulations in a series of mononuclear dysprosium complexes, [DyLz_2_(*o*-vanilin)_2_]·X·solvent (Lz = 6-pyridin-2-yl-[1,3,5]triazine-2,4-diamine; X = Br^–^ (**1**), NO_3_^–^ (**2**), CF_3_SO_3_^–^ (**3**)), were realized by changing the nature of the counter-anion. The Dy^III^ ions in all complexes are eight-coordinate and in approximate *D*_4d_ symmetry environments. The magnetic relaxation and anisotropy of these complexes were systematically investigated, both experimentally and from *ab initio* calculations. All complexes exhibit excellent single-molecule magnetic behavior. Remarkably, magneto-structural studies show that the rotation of the coordinating plane of the square-antiprismatic environment in complex **2** induces a magnetic relaxation path through higher excited states, yielding a high anisotropy barrier of 615 K (696 K for a diluted sample). Additionally, obvious opening of the hysteresis loop is observed up to 7 K, which is the highest blocking temperature ever reported for dysprosium single-molecule magnets.

## Abstract 2

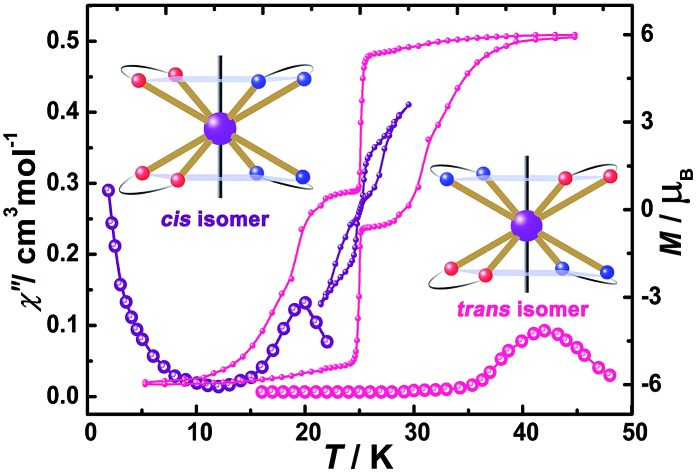
The rotation of the coordinating plane of the square-antiprismatic environment induces a magnetic relaxation path through higher excited states, offering a new way to modulate the geometries of lanthanides to facilitate magnetic relaxation climbing up to higher energy levels.

## Introduction

In the quest of the ultimate miniaturization of magnets, fascinating investigations have converged on lanthanide single-molecule magnets (SMMs)^1^ with promising applications in high-density information storage and molecular spintronics.^2^ Recent results have shown the potential of the element dysprosium in the design of SMMs for future applications due to its doubly degenerate ground state and large intrinsic anisotropy.^3^ However, large effective barriers of SMMs have been identified in terbium-phthalocyanine derivatives^4^ and an utmost blocking temperature was detected in a N_2_^3–^ radical-bridged terbium complex,^5^ suggesting terbium to be potentially superior to dysprosium in the design of robust SMMs with high effective barriers and blocking temperatures. Although a remarkable blocking temperature of 7 K was extracted from the polymetallic Dy@Y_4_K_2_ complex,^6^ it is still a formidable challenge to push the frontiers to higher temperature regimes, due to the fast quantum tunneling of magnetization (QTM) concomitant with the intricate coordination geometry.^7^ Recently, a NCN-pincer ligand dysprosium complex^8^ and an equatorially coordinated erbium mononuclear single-molecule magnet,^9^ reported by our group, represent successful examples that simplifying the ligand field facilitates relaxations *via* higher excited states, thus opening new perspectives for enhancing the anisotropy of excited doublets through lowering the coordination number.3a,3c

Aside from low-coordinate systems, high symmetry cases, such as *D*_4d_ and *D*_5h_, have been widely investigated previously,4a,^10^ in which QTM can be suppressed by tuning the local symmetry.10*i* Among the *D*_5h_ symmetry dysprosium SMMs reported to date, DyM_2_ (M = Zn, Fe) complexes represent the most successful enhancement of the magnetic blocking barrier, in which the axial crystal field induces large anisotropic properties.10i,10j However, the situation becomes more complicated for *D*_4d_ symmetry dysprosium complexes. For example, in the polyoxometalate4a,4c and phthalocyanine sandwich-type10*a* families, where the lanthanide ions possess an almost perfect *D*_4d_ coordination environment,^11^ the SMM properties of dysprosium complexes are less prominent when compared with their terbium and erbium analogues, while the distorted *D*_4d_ coordination polyhedron in the β-diketonate series gives rise to strong Ising ground states, leading to significant relaxation blockages for dysprosium derivatives.10b,10g It seems that not only the coordination geometry, but also the coordination environment, such as the type of coordinating atoms, the identity and nature of the ligand and *cis*–*trans* isomerism, could influence the relaxation behavior.

**Scheme 1:**
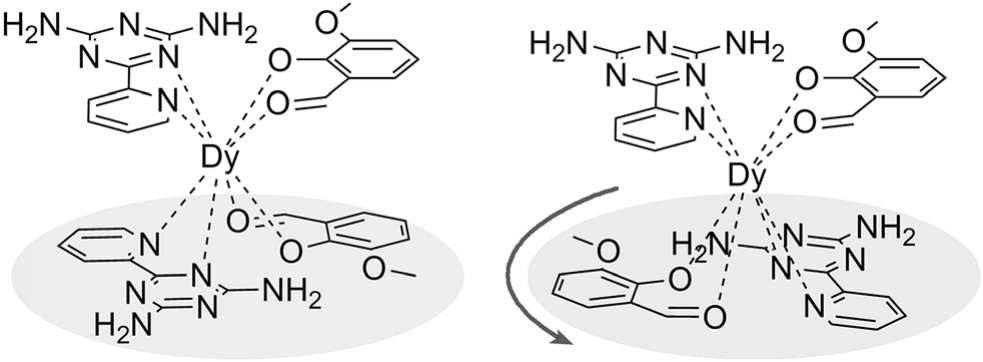
Schematic drawing of complexes **1**, **3** (left) and **2** (right).

With this in mind, we intend to probe the effect of coordination environments on the relaxation dynamics of lanthanide SMMs. Herein, a series of mononuclear dysprosium complexes of the formula [DyLz_2_(*o*-vanilin)_2_]·X·solvent (Lz = 6-pyridin-2-yl-[1,3,5]triazine-2,4-diamine; X = Br^–^ (**1**), NO_3_^–^ (**2**), CF_3_SO_3_^–^ (**3**)), with the metal ions in a distorted *D*_4d_ coordination environment (Scheme 1), were synthesized and structurally and magnetically characterized. *Ab initio* calculations were also performed in order to rationalize the magnetic behavior of the above-mentioned complexes. The change of the counter-anion results in great differences in the coordination environment and dramatically alters the relaxation behavior. Among these stable and simple complexes, complex **2** exhibits slow magnetic relaxation at temperatures approaching 50 K and the thermal energy barrier for the reversal of magnetization reaches 696 K, which is the largest observed yet for mononuclear dysprosium SMMs. Furthermore, the opening of the hysteresis loop up to 7 K using the sweep rate accessible with a conventional magnetometer is also remarkable.

## Experimental

All chemicals and solvents were commercially obtained and used as received without any further purification. IR spectra were recorded with a Perkin-Elmer Fourier transform infrared spectrophotometer. Elemental analysis for C, H, N and S was carried out on a Perkin-Elmer 2400 analyzer. All doped samples were analyzed using Inductively Coupled Plasma Optical Emission Spectrometry (ICP OES). The ligand Lz (2,4-diamino-6-pyridyl-1,3,5-triazine) was prepared according to a previously published method^12^ under ambient conditions. A mixture of 2-cyanopyridine (0.1 mol), dicyandiamide (0.125 mol), potassium hydroxide (0.02 mol) and 1,2-dimethoxyethane (0.62 mol) was refluxed for 4 h. After cooling, the contents were poured into water, the precipitate was collected by filtration and dried under vacuum. IR (cm^–1^): 3462 (m), 3393 (m), 3273 (b), 3130 (b), 1611 (m), 1536 (m), 1394 (s), 1253 (m), 993 (m), 830 (m), 792 (s), 687 (w).

### Syntheses of complexes **1–3**

All complexes were prepared by similar procedures; therefore only the synthesis of complex **1** is described here in detail. The reaction of DyBr_3_·6H_2_O (0.15 mmol) with Lz (0.1 mmol) and *o*-vanilin (0.1 mmol) in the presence of triethylamine (0.1 mmol), with 5 : 5 ml methanol/acetonitrile as the media, produced yellow crystals of **1** after 7 days. Anal. calcd for **1** (C_34_H_34_BrDyN_13_O_6.5_, MW = 971.15): C, 42.01%; H, 3.50%; N, 18.74%. Found: C, 42.12%; H, 3.61%; N, 18.69%. For the synthesis of **2** and **3**, Dy(NO_3_)_3_·5H_2_O and Dy(CF_3_SO_3_)_3_·6H_2_O were used to replace DyBr_3_·6H_2_O with methanol (15 ml) as the solvent. Anal. calcd for **2** (C_32_H_30_DyN_13_O_9_, MW = 903.19): C, 42.52%; H, 3.32%; N, 20.15%. Found: C, 41.93%; H, 3.51%; N, 20.26%. Anal. calcd for **3** (C_33_H_30_DyF_3_N_12_O_11_S, MW = 1022.26): C, 38.73%; H, 2.93%; N, 16.43%; S, 3.13%. Found: C, 38.55%; H, 3.06%; N, 15.91%; S, 3.20%. Doping of complex **2** was performed by adding Dy(NO_3_)_3_·5H_2_O and Y(NO_3_)_3_·5H_2_O together (with ratios of 1 : 1, 1 : 20 and 1 : 50) in the reaction. ICP OES analysis found the components of [Dy_0.671_Y_0.329_Lz_2_(*o*-vanilin)_2_]·NO_3_, [Dy_0.076_Y_0.924_Lz_2_(*o*-vanilin)_2_]·NO_3_ and [Dy_0.024_Y_0.976_Lz_2_(*o*-vanilin)_2_]·NO_3_ corresponding to the 2 : 1, 1 : 12 and 1 : 39 samples.

### Crystallography

Single-crystal X-ray data of the title complexes were collected on a Bruker Apex II CCD diffractometer equipped with graphite monochromated Mo-Kα radiation (*λ* = 0.71073 Å) at 293(2) K. The structures were solved by direct methods and refined by full-matrix least-squares methods on *F*^2^ using SHELXTL-97.^13^ All non-hydrogen atoms were determined from the difference Fourier maps and refined anisotropically. Hydrogen atoms were introduced in calculated positions and refined with fixed geometry with respect to their carrier atoms. Crystallographic data are listed in Table S1.†

### Magnetic measurements

Magnetic susceptibility measurements were recorded on a Quantum Design MPMS-XL7 SQUID magnetometer equipped with a 7 T magnet. The variable-temperature magnetization was measured in the temperature range of 1.9–300 K with an external magnetic field of 1000 Oe. The dynamics of the magnetization were investigated from the ac susceptibility measurements in the zero static fields and a 3.0 Oe ac oscillating field. Diamagnetic corrections were made with the Pascal's constants^14^ for all the constituent atoms, as well as the contributions of the sample holder.

### Computational details

Wavefunction-based calculations were carried out on the X-ray structures of **1**, **2** and **3** using the SA-CASSCF/RASSI-SO approach as implemented in MOLCAS 8.0,^15^ in which the relativistic effects are treated by means of the Douglas–Kroll Hamiltonian in a two step scheme. First, the scalar terms are included in the basis-set generation and are used to determine the spin-free states in the Complete Active Space Self-Consistent Field (CASSCF) method.^16^ Next, spin–orbit coupling is added within the Restricted Active Space State-Interaction (RASSI-SO) method,^17^ in which the spin-free states serve as basis states. The resulting energies and wave functions are finally used to compute the magnetic properties (*i.e.* magnetization and 2K-magnetic susceptibility curves, anisotropy tensors of the low-energy states of the system, as well as the associated wave functions in term of *M*_J_ eigenstates) using the pseudo-spin *S* = 1/2 approximation as defined in the SINGLE_ANISO routine.^18^ The Cholesky decomposition is used when computing bielectronic integrals.^19^ The active space of the CASSCF calculations consisted of the nine 4f electrons of the Dy^III^ ion spanning the seven 4f orbitals, *i.e.* CAS(9,7)SCF. The state-averaged CASSCF calculations were performed for all the 21 sextet roots, all the 224 quadruplet roots and 300 out of the 490 doublet roots, due to software limitations. In the RASSI-SO calculation, the 21 sextet roots were allowed to mix through spin–orbit coupling with the first 128 quadruplet roots and the first 107 doublet roots. All atoms were described by ANO-type basis sets from the ANO-RCC library of MOLCAS. The following contractions were used: [8s7p4d3f2g1h] for the Dy^III^ ion, [4s3p2d] for the four N and the four O atoms of the first coordination sphere, [3s2p1d] for the remaining N and O atoms and all C atoms, and [2s] for the H atoms.

## Results and discussion

**Fig. 1:**
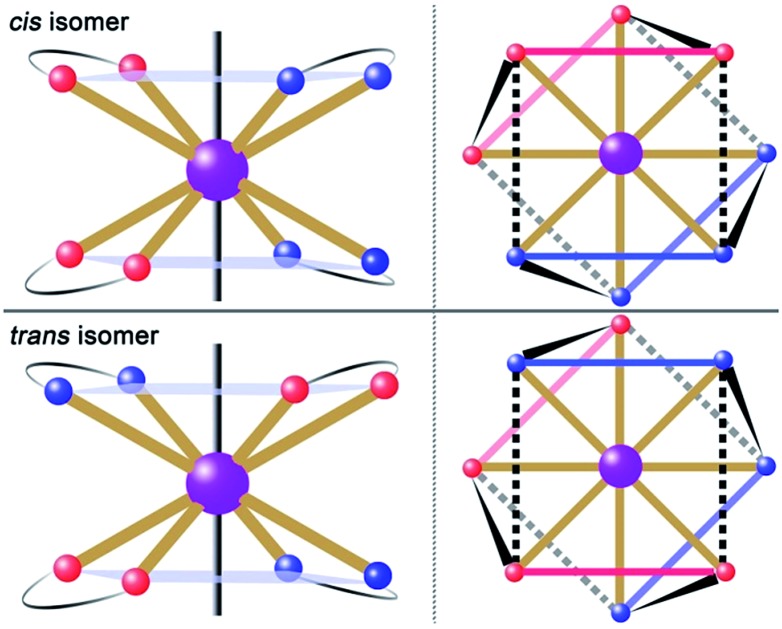
Schematic drawing of absolute configurations with top views (right) for the *cis* (top) and *trans* (bottom) configurations of the [DyLz_2_(*o*-vanilin)_2_]^+^ units.

**Fig. 2:**
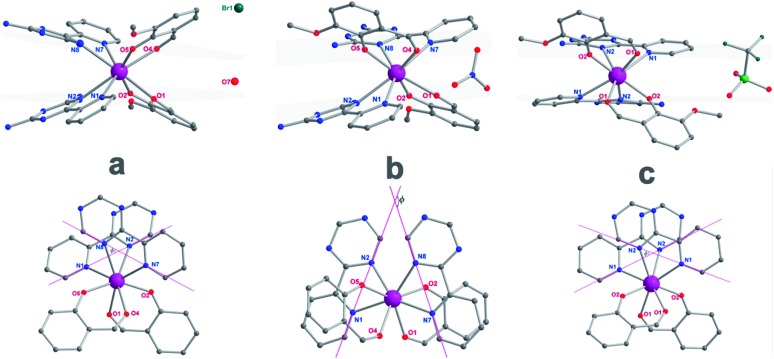
The side view (top) and top view (bottom) of complexes **1** (a), **2** (b) and **3** (c) with pink, dark, blue and red spheres representing Dy, C, N, and O, respectively; the grey planes (top) represent the upper and lower coordination planes and the *Φ* value (bottom) was defined as the space angle between the two Lz ligands. The hydrogen atoms and solvents have been omitted for clarity.

The reaction of the dysprosium salt (0.15 mmol) with Lz (0.1 mmol) and *o*-vanilin (0.1 mmol) in the presence of triethylamine (0.1 mmol), with 5 : 5 ml methanol/acetonitrile as the reaction media leads to the formation of mononuclear dysprosium complexes, [DyLz_2_(*o*-vanilin)_2_]·X·solvent (Lz = 6-pyridin-2-yl-[1,3,5]triazine-2,4-diamine; X = Br^–^ (**1**), NO_3_^–^ (**2**), CF_3_SO_3_^–^ (**3**)). The structures of **1**, **2** and **3** are depicted in Fig. S1–S3.† In all three complexes, the Dy^III^ ion is in a N_4_O_4_ square-antiprismatic environment (Fig. 1 and Table S2†), where the four nitrogen atoms come from the two Lz ligands, and the four oxygen atoms come from the two *o*-vanilin ligands. The latter ligands are arranged in planes in between which the Dy^III^ ion is sandwiched. Each plane consists of one Lz and one *o*-vanilin ligand, the relative orientation of which is mainly driven by hydrogen bonding between the hydroxy oxygen of the *o*-vanilin ligand and one hydrogen atom in the triazine part of the Lz ligand (O···H distances are 2.07 Å and 2.17 Å for **1**, 2.11 Å and 2.14 Å for **2**, and 2.05 Å for **3**). In all three complexes, the positive charge is balanced by one counter-anion, namely Br^–^ in **1**, NO_3_^–^ in **2** and CF_3_SO_3_^–^ in **3**. Although **1**, **2** and **3** have similar ligand sets, a closer look at the structural features reveals significant ligand rotation and considerable distortion from the *D*_4d_ symmetry, which is most likely induced by the change of counter-anions. In order to evaluate these differences, the *α* angle between the pseudo *S*_8_ axis and the Dy–L directions, the *Φ* space angle between the two Lz ligands, as well as the *θ* angle between the upper and lower mean planes, were investigated (Fig. 2 and S4†) and the values are listed in Table S3.† A relatively small *α* angle is found in **2** (56.5°) with respect to **1** and **3** (57.0° in both systems), indicating an axial extension of the coordinating environment in **2**. This can be explained by the insertion of the NO_3_^–^ counter-anion between the coordination planes in **2**, while in **1** and **3** the counter-anions reside outside these planes and relatively far from the molecular unit. For the *Φ* angle, small values are found in **1** and **3** (53.8° and 47.1°, respectively) and are characteristic of the two Lz ligands being in the *cis* position relative to each other, while the much larger value found in **2** (140.9°) is characteristic of the two Lz ligands being in the *trans* position (Fig. 1). Finally, the *θ* angles for **1**, **2** and **3** are 6.1°, 4.8° and 2.6°, respectively, suggesting that π-stacking interactions between the Lz ligands occur more preferably in **3** than in **1**, which is supported by the short inter-planar distance (2.61 Å) and π-stacking distance between the two triazinyl centers of the ligand Lz (3.56 Å) found in **3** (Fig. S3†). With **2** being in the *trans* configuration, no π-stacking can occur between the two Lz ligands due to the large *Φ* angle. In **1** and **2**, crystal packing is governed by both π-stacking interactions between the Lz ligands of neighboring molecules, and H-bonding between the triazine parts of the Lz ligands not involved in π-stacking interactions. In **3**, molecular units are related only through π-stacking interactions between the Lz or the *o*-vanilin ligands of neighboring molecules. The shortest intermolecular Dy···Dy distances are 7.9, 7.7 and 7.5 Å for **1**, **2** and **3**, respectively, suggesting the existence of weak dipolar interactions. Such significant changes to the structure upon changing the counter-ions are rare in SMMs systems, but are most likely responsible for the alteration in the magnetic relaxation properties of these complexes7b,7c,^20^ (see below).

### Magnetic properties

**Fig. 3:**
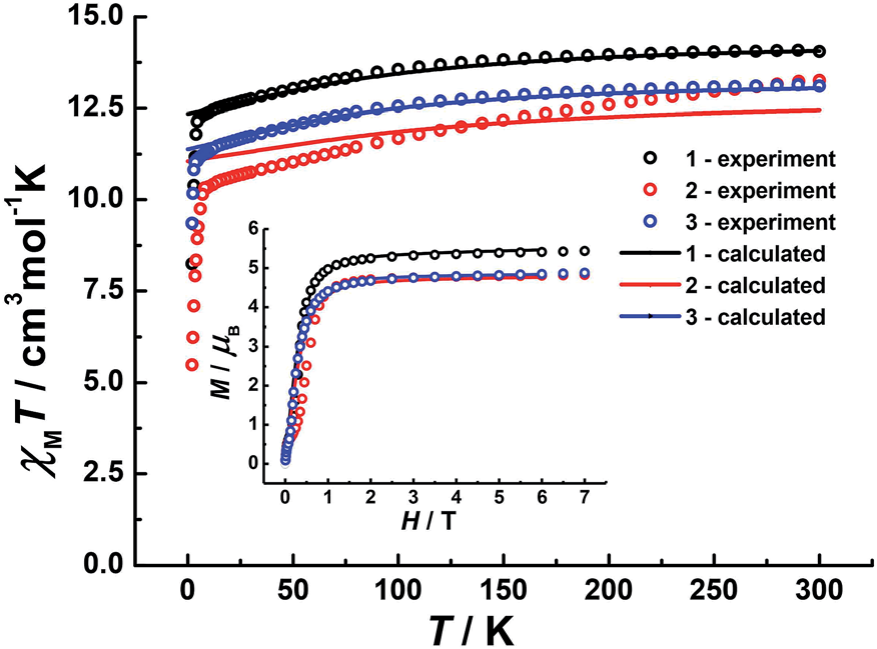
Temperature dependence of *χ*_M_*T* products at 1 kOe, between 2 and 300 K for **1** (dark), **2** (red) and **3** (blue). Inset: plots of *M*–*H* for **1**, **2** and **3** at 2 K. The solid lines correspond to *ab initio* calculations.

Direct-current (dc) magnetic susceptibilities were investigated for **1–3** under a 1 kOe applied field from 2 to 300 K (Fig. 3). The room-temperature *χ*_M_*T* value of 14.1 cm^3^ K mol^–1^ for **1** is close to the expected value of 14.17 cm^3^ K mol^–1^ for the Dy^III^ single ion. The *χ*_M_*T* values of 13.3 cm^3^ K mol^–1^ and 13.1 cm^3^ K mol^–1^ for **2** and **3** are slightly smaller than the theoretical value, which might be ascribed to the fact that the ground Russell–Saunders multiplet, having been split by the crystal field, is not equally populated even at room temperature, since *χ*_M_*T* keeps increasing upon warming near room temperature.^21^ Upon cooling, *χ*_M_*T* decreases slowly down to 4 K, 7 K and 3 K, reaching, after a sudden drop, values of 8.2, 5.5 and 9.3 cm^3^ K mol^–1^ at 2.0 K for **1**, **2** and **3**, respectively. This sudden drop is indicative of magnetic blocking.^22^ At 1.9 K, the molar magnetization (*M*) *vs. T* curves for **1–3** (Fig. S5–S7†) saturate rapidly at about 5 *μ*_B_, which is consistent with a pure Ising ground state. *Ab initio* calculations confirm the strong axiality of the ground states in **1–3** with large *g*_Z_ values (19.72, 19.80 and 19.68 for **1**, **2** and **3**, respectively) close to the expected value of 20.00 for a pure *M*_J_ = ±15/2 eigenstate. Wave-function analysis also confirms greater than 80% percentages of the *M*_J_ = ±15/2 eigenstate in **1–3**.

**Fig. 4:**
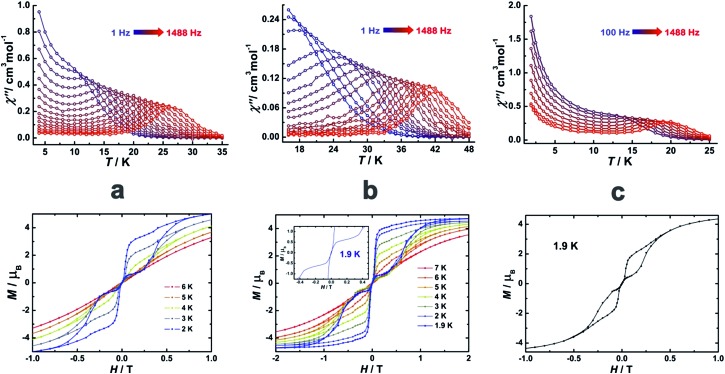
Temperature-dependent out-of-phase (*χ*′′) magnetic susceptibilities (top) and magnetic hysteresis (bottom) for **1** (a), **2** (b) and **3** (c) at indicated temperatures.

**Fig. 5:**
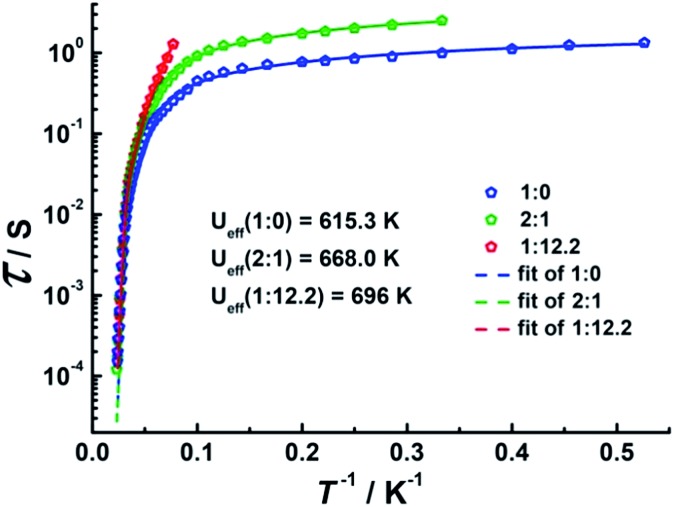
Plots of *τ vs. T*^–1^ at *H*_dc_ = 0 Oe for diluted samples with Dy : Y ratios of 1 : 0, 2 : 1 and 1 : 12.2.

To investigate the SMM behavior of **1–3**, alternating-current (ac) magnetic susceptibility measurements were also performed under zero dc fields (Fig. 4). Temperature-dependent in-phase (*χ*′) and out-of-phase (*χ*′′) magnetic susceptibility signals at 1488 Hz for **1–3** exhibit peaks at 27 K, 42 K and 20 K, respectively. Upon cooling, a new tail peak appears below 2.5 K in the *χ*′ and *χ*′′ signals of **1** and **2**, while a rapid increase is observed below 10 K for **3**. This rapid increase in the low temperature region could be attributed to quantum tunneling effects at zero dc field, which is very common in 4f SMMs.^23^ Frequency-dependent susceptibility data were collected in the range of 1–1488 Hz under zero applied dc field (Fig. S8–S10†). For **3**, as the temperature is lowered, the maximum peak in the out-of-phase *χ*′′ signal is shifted toward a lower frequency until 8 K, beyond which the same frequency was maintained, confirming the presence of the classical quantum regime (Fig. S10†). Similar behavior was observed for **1** and **2** below 2 K and 4 K, respectively, indicating slow relaxation of the magnetization associated with SMM behavior. To evaluate the energy barrier, relaxation times were extracted from the maxima of the out-of-phase signal (Fig. S11–S13†). The Arrhenius fits yield effective energy barriers of *U*_eff_ = 221, 615 and 120 K, for **1**, **2** and **3**, respectively. It is noteworthy that the energy barrier for complex **2** is the highest known for a mononuclear dysprosium-based SMM. To reduce the dipole–dipole interactions between the magnetic centers and slow down relaxation, diluted samples were prepared. Magnetic dilution studies for **2** (Fig. 5) show great enhancement of the magnetic relaxation, giving a relaxation time as long as 2.5 s. The extracted effective energy barrier reaches 696 K (484 cm^–1^) with a *τ*_0_ = 5.7(5) × 10^–11^ s. The Cole–Cole plots of *χ*′′ *versus χ*′ display semi-circular profiles and are fitted to a generalized Debye model (Fig. S14–S16†).^24^ The values of the *α* parameter are relatively large (*α* ≤ 0.32, 0.34 and 0.23 for **1**, **2** and **3**, respectively), indicating a relatively wide distribution of the relaxation times, and thus multiple pathways for spin reversal.^25^ Thus, all plots were fitted to multiple relaxation processes, requiring Orbach,^26^ Raman, and quantum-tunnelling processes.^27^ The obtained values of *τ*_0_ and *U*_eff_ are listed in Table S4.†

**Fig. 6:**
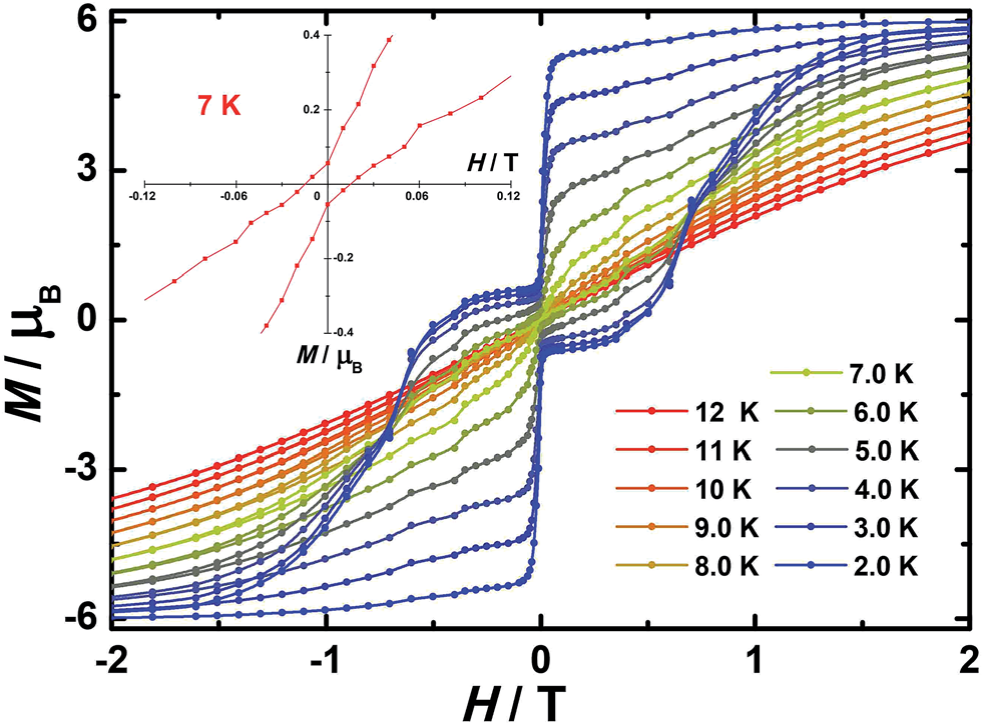
Magnetic hysteresis for diluted sample of **2** (Dy : Y = 1 : 39) with clear opening of the hysteresis loop under sweep rate accessible with a conventional magnetometer. Inset: zoomed in hysteresis loop.

In order to investigate the blocking of magnetization, magnetic hysteresis measurements were performed on **1–3** (Fig. 4 and S17†). The magnetic hysteresis of complex **1** displays a clear butterfly shape hysteresis with openings up to 6 K at *H* ≠ 0. Similar magnetic hysteresis was obtained for complex **3**, though with a smaller opening (*H* ≠ 0). In contrast to **1** and **3**, complex **2** displays distinct butterfly shape hysteresis. Herein, the hysteresis loops remain open until 7 K with a much larger opening near zero-field. More interestingly, non-coincidence was observed in the *M vs. H* curves at *H* = 0 (Fig. 4 inset), suggesting a potential remnant magnetization and coercive field. In order to enlarge the opening gap, magnetic hysteresis measurements were also conducted on the diluted samples. As expected, significant improvements were observed on the diluted sample with a Dy : Y ratio of 1 : 39, with a clear opening now observed up to 7 K at *H* = 0, and remnant magnetization and coercive field of 0.75 *μ*_B_ and 0.46 T, respectively, at 2.0 K (Fig. 6 and S18–S22†).

All complexes presented herein show large energy barriers, which is rare among the dysprosium-SMM family, and is most likely due to the unique *D*_4d_ environment at the Dy^III^ center, as well documented^28^ for β-diketonate Dy^III^ derivatives (Table S5†). Additionally, it is known that the use of large aromatic auxiliary groups results in systems with highly uniaxial magnetic anisotropies and high thermal barriers.10*f* Therefore, the application of large aromatic aldehyde (*o*-vanilin) and triazine analogues (Lz) is expected to significantly influence the energy spectrum and magnetic anisotropy of the low-lying states of Dy^III^, and thus lead to efficient Dy-based SMMs, which is indeed the case here. However, the different environment geometries and subsequent various magnetic behaviors induced by the change of counter-anions in our system with the same set of ligands are remarkable. Notably, the energy barrier observed in complex **2** is extraordinary larger than those in **1** and **3**, which might be ascribed to the rotation of the coordinating plane of the square-antiprismatic environment.

**Fig. 7:**
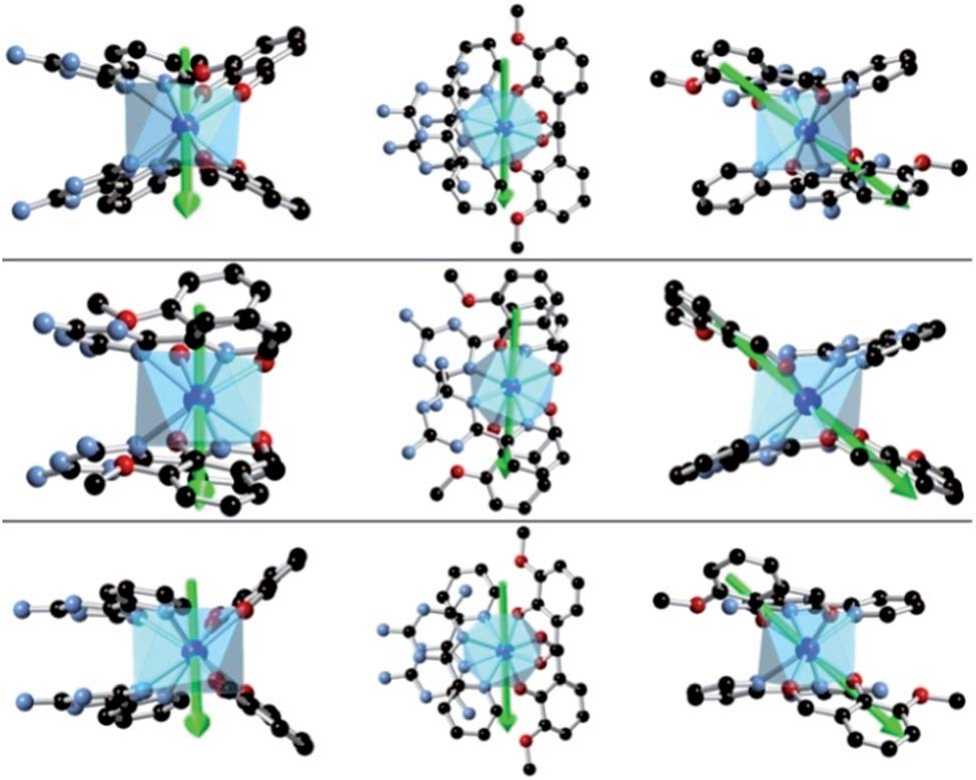
*Ab initio* magnetic easy-axes (in various orientations) of the ground states of **1** (top), **2** (middle) and **3** (bottom).

**Fig. 8:**
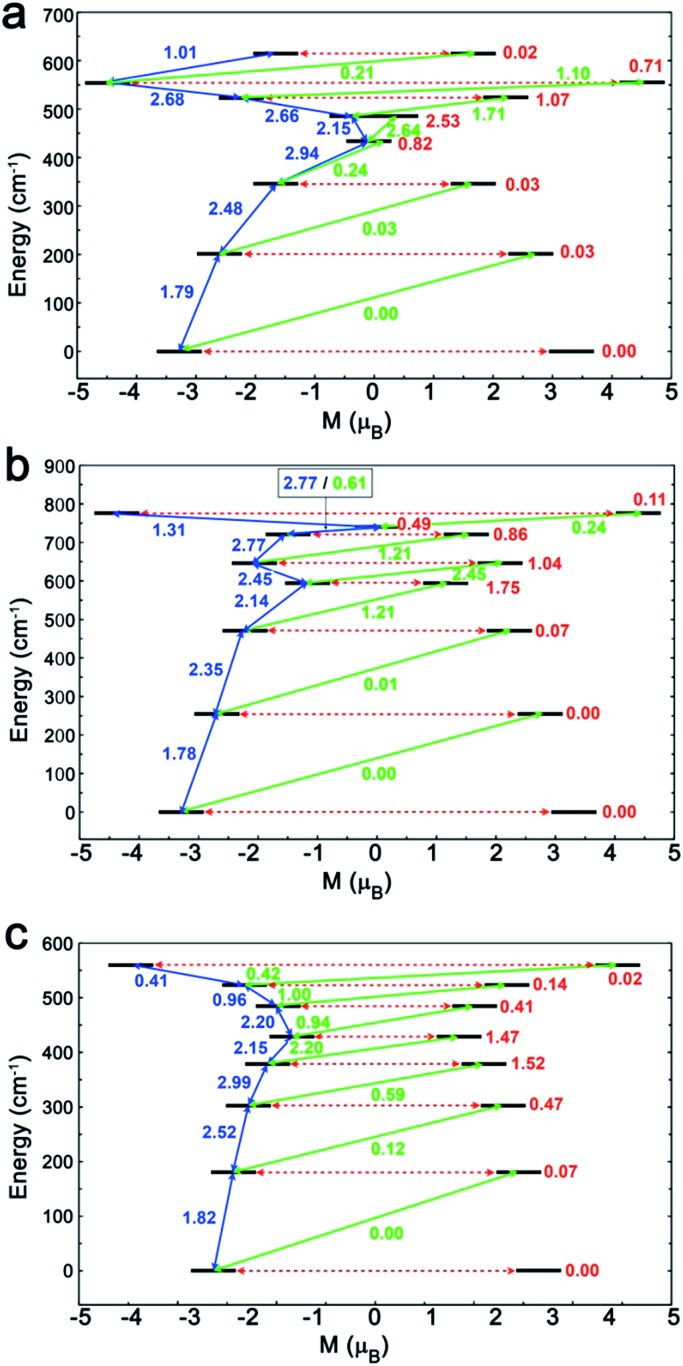
The magnetization blocking barriers and relaxation pathways with highest probability in complexes **1** (a), **2** (b) and **3** (c).

To investigate this trend further, the transition moments between all Kramer doublets from the ^6^H_15/2_ ground multiplet of Dy^III^ are computed from *ab initio* for all complexes (Fig. 7, 8 and S23†). In **1**, the most probable pathway for relaxation is found to go, at least, through the 3rd excited state, reaching an energy barrier of approximately 430 cm^–1^ (620 K). This value is much higher than that obtained from ac measurements (221 K), but it has to be kept in mind that this kind of calculation does not account for all possible relaxation mechanisms (in particular, indirect mechanisms are not accounted for), and relies on several approximations.3*a* However, the latter pathway is supported by the magnetic anisotropy features of the low-lying excited states. Indeed, the ground state has strongly axial magnetic anisotropy with zero transversal components (Table S6†). The same goes for the 1st and 2nd excited states, for which the magnetic anisotropy is strongly axial (very small transversal components), with small deviations in the orientation of the associated magnetic easy-axis (Table S6†). On the contrary, the 3rd and higher excited states have magnetic anisotropies with large transversal components, inducing quantum tunneling, and thus short-cutting the direct relaxation process. In **3**, the situation is almost the same, except that large transversal components already appear at the 2nd excited state (Table S6†), through which calculations showed a non-zero probability of transition. The associated energy barrier is approximately 300 cm^–1^ (430 K). Here again, the computed value is much higher than that from ac measurements (120 K), but still smaller than that of **1**, which is in good agreement with the experimental tendency. Finally, for **2**, calculations evidence a relaxation pathway going through the 3rd excited state, leading to an energy barrier of approx. 600 cm^–1^ (860 K). This pathway is supported by the high axiality of the magnetic anisotropy of the three lower states and the small angular deviation between the associated magnetic easy-axis directions, while for the 3rd excited state, the transversal components become very large, with a large deviation to the direction of the ground state easy-axis (Table S6†). The tendency with respect to the values obtained from ac measurements is respected for the whole series, since the energy barrier in **2** is much larger than those of **1** and **3**. In the end, it appears that magnetic relaxation goes more or less through the same states (*i.e.* the 2nd or 3rd excited states) in all complexes, suggesting that the main factor responsible for the difference in energy barriers in **1–3** is the total energy splitting of the ^6^H_15/2_ ground multiplet, which itself depends on the structural features of each complex, and most likely on the *cis* or *trans* configuration of the Lz ligands. Indeed, in the *cis* configuration, contrary to the *trans* configuration, π-stacking interactions are operative and contribute towards reducing the energy splitting of the ^6^H_15/2_ ground multiplet. This explains why the splitting is much larger in **2** (*trans* configuration) than in **1** and **3** (*cis* configuration). In more detail, this also explains why the energy splitting of **1** is larger than that of **3**, since π-stacking interactions are much more effective in **3** than in **1**, thus having a larger stabilizing effect. Additionally, the axial extension in **2** might also, to some extent, be responsible for the associated larger splitting, as well as the exact *C*_2_ symmetry held by **3** (Fig. S24 and S25†) might be responsible for further stabilization of its energy splitting with respect to **1**.

## Conclusions

In conclusion, we have structurally isolated three dysprosium SMMs with similar square-antiprismatic environments (*D*_4d_) but discriminative counter-anions. The distortions in the *D*_4d_ symmetry environment led to large energy gaps between the ground and first excited states, and strong axial anisotropies for complexes **1–3**. Upon changing the counter-anion, significant differences in the structure and thus magnetic behavior were observed: for instance, when going from **3** to **1**, the π–π stacking interactions between the Lz ligands lead to axial constriction in the environment geometry, from which fast quantum tunneling arises because the symmetry is lowered; proper rotation of one coordinating plane, as well as relative axial extension in complex **2** with respect to complex **3**, allows magnetic relaxation to pass through higher excited states, due to an increase in molecular symmetry. *Ab initio* calculations substantiate the diversity of the magnetic behaviors in complexes **1–3** and suggest that the ground states are almost isolated, magnetically speaking. Notably, the efficient magnetic relaxation pathways of complex **2** probably go through the fourth and fifth Kramer doublet states. This conforms a high anisotropy barrier of 696 K (484 cm^–1^) and magnetic blocking up to 7 K. This work offers a new way to modulate the geometries of lanthanides in order to facilitate magnetic relaxation climbing up to higher energy levels.

## Supplementary Material

Supplementary information

Crystal structure data
