# Supplementary material for: Cis–trans isomerism modulates the magnetic relaxation of dysprosium single-molecule magnets
Source: Chem Sci. 2016 Feb 16;7(6):3632–9. doi: 10.1039/c5sc04510j (PMC6008742; doi:10.1039/c5sc04510j)
Supplement: Supplementary file 1 [file SC-007-C5SC04510J-s001.pdf]

Electronic Supplementary Material (ESI) for Chemical Science.
This journal is © The Royal Society of Chemistry 2016

Cis-Trans Isomerism Modulates the Magnetic Relaxation of Dysprosium Single-Molecule Magnets

Jianfeng Wu,[a,c] *Julie Jung,*[b] *Peng Zhang,*[a] *Haixia Zhang,*[a] *Jinkui Tang,**[a] *and Boris Le Guennic**[b]

Table S1. Crystallographic data for complexes **1**–**3**.

	**1**	**2**	**3**
Formula	$C_{34}H_{34}BrDyN_{13}O_{6.5}$	$C_{32}H_{30}DyN_{13}O_9$	$C_{33}H_{30}DyF_3N_{12}O_{11}S$
FW, g·mol^{-1}	971.15	903.19	1022.26
crystal system	Triclinic	Monoclinic	Monoclinic
space group	*P* -1	*C* 2/*c*	*C* 2/*c*
T, K	293(2)	296(2)	296(2)
λ, Å	0.71073	0.71073	0.71073
a, Å	10.5922(6)	30.250(5)	20.791(2)
b, Å	12.3446(8)	17.599(3)	18.7611(18)
c, Å	15.3053(9)	20.537(3)	13.6144(14)
α, °	107.3370(10)	90	90
β, °	90.5590(10)	129.531(11)	129.032(2)
γ, °	90.5540(10)	90	90
V, Å^3	1910.1(2)	8433(2)	4125.1(7)
Z	2	8	4
ρ_{calcd}, g·cm^{-3}	1.689	1.1	1.646
μ (Mo, Kα), mm^{-1}	3.066	1.834	1.947
reflns collected	12191	26371	11904
R_1[a)], wR_2 ($I \geq 2\sigma(I)$)[b)]	0.0582, 0.1016	0.0482, 0.1354	0.0462, 0.1242
R_1, wR_2 (all data)	0.0995, 0.1207	0.0739, 0.1469	0.0573, 0.1305
CCDC number	1041137	1041138	1041139

[a)] $R_1 = \sum||F_o|-|F_c||/\sum|F_o|$, [b)] $wR_2 = [\sum w(F_o^2-F_c^2)^2/\sum w(F_o^2)^2]^{1/2}$

Table S2. *CShM* values calculated by *SHAPE* 2.1[1] for **1**, **2** and **3**.

Central atom	Coordination Geometry	Complex **1**	Complex **2**	Complex **3**
Dy	Cube (O_h)	8.104	10.496	10.656
	Square antiprism (D_{4d})	1.018	0.855	0.700
	Triangular dodecahedron (D_{2d})	1.957	2.536	2.433
	Bi-augmentedtrigonal prism (C_{2v})	2.661	2.576	2.589

Table S3. Selected bond distances (Å), angles (º) and D_{4d} distortion parameters for complexes **1-3**.

	**1**	**2**		**3**
Dy···Dy	7.924	7.672	Dy···Dy	7.461
Dy(1)-O(5)	2.216(5)	2.238(4)	Dy(1)-O(2)	2.207(4)
Dy(1)-O(2)	2.216(5)	2.254(4)	Dy(1)-O(2)	2.207(4)
Dy(1)-O(1)	2.383(6)	2.373(5)	Dy(1)-O(1)	2.373(5)
Dy(1)-O(4)	2.393(6)	2.383(5)	Dy(1)-O(1)	2.373(5)
Dy(1)-N(1)	2.494(6)	2.500(5)	Dy(1)-N(1)	2.493(5)
Dy(1)-N(7)	2.486(6)	2.513(5)	Dy(1)-N(1)	2.493(5)
Dy(1)-N(8)	2.570(7)	2.526(5)	Dy(1)-N(2)	2.585(5)
Dy(1)-N(2)	2.590(7)	2.551(5)	Dy(1)-N(2)	2.585(5)
$\Phi^{\circ a)}$	53.821	140.854	Φ°	47.098
$\alpha^{\circ b)}$	57.016	56.542	α°	57.013
$\theta^{\circ c)}$	6.128	4.841	θ°	2.59
$dpp^{d)}$	2.6386	2.6499	dpp	2.6076

[a)] The angle defined as the space angle between two Lz ligands, [b)] The average angle between the pseudo S_8 axis and a Dy–L direction. [c)] The angle between the upper and lower planes containing the coordinated atoms. [d)] The distance between the upper and lower planes.

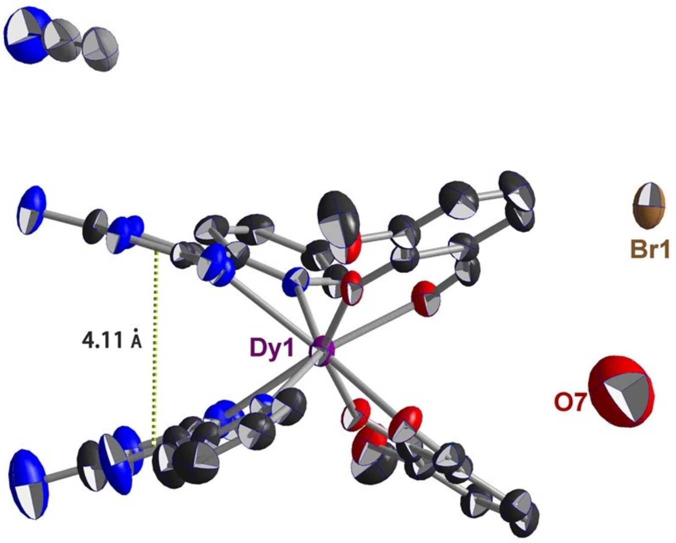

Fig. S1 ORTEP view of complex **1**; the hydrogen atoms have been omitted for clarity. The green dashed line represents π-stacking distance between the two triazinyl centers of ligand Lz.

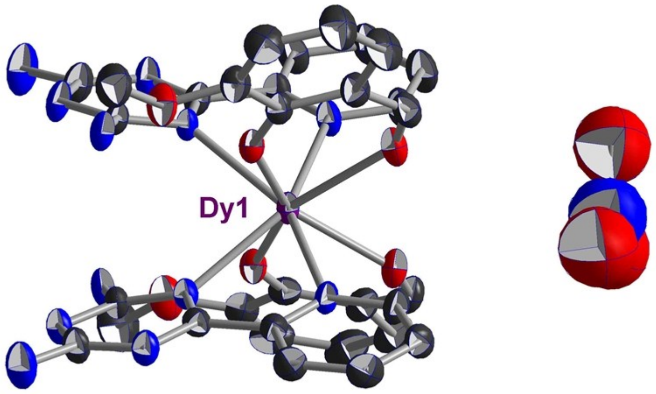

Fig. S2 ORTEP view of complex **2**; the hydrogen atoms have been omitted for clarity.

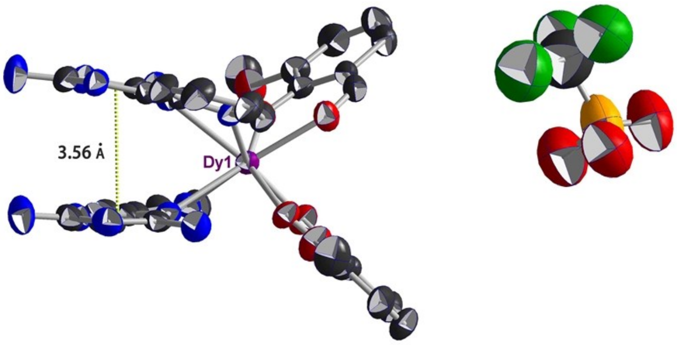

Fig. S3 ORTEP view of complex **3**; the hydrogen atoms have been omitted for clarity. The green dashed line represents π-stacking distance between the two triazinyl centers of ligand Lz.

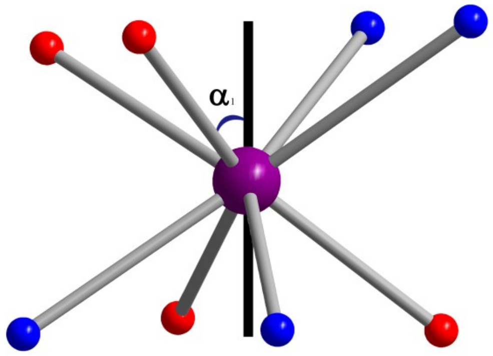

Fig. S4 D_{4d} coordination mode with the α angle superimposed.

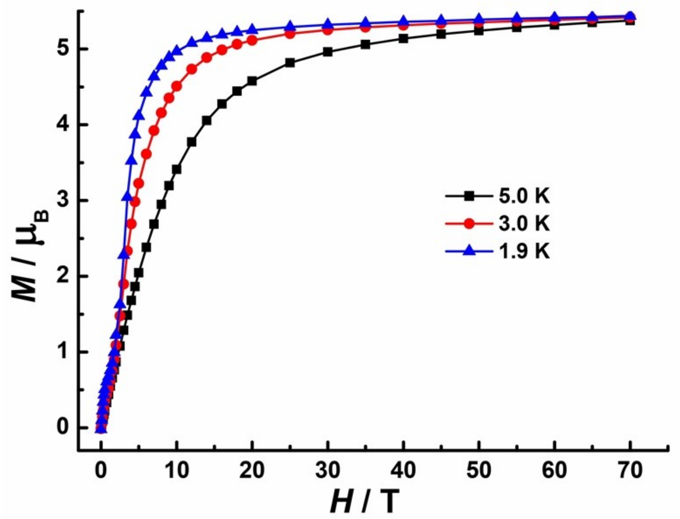

Fig. S5 Molar magnetization (*M*) *vs.* magnetic field (*H*) for **1** at 1.9, 3.0, and 5.0 K.

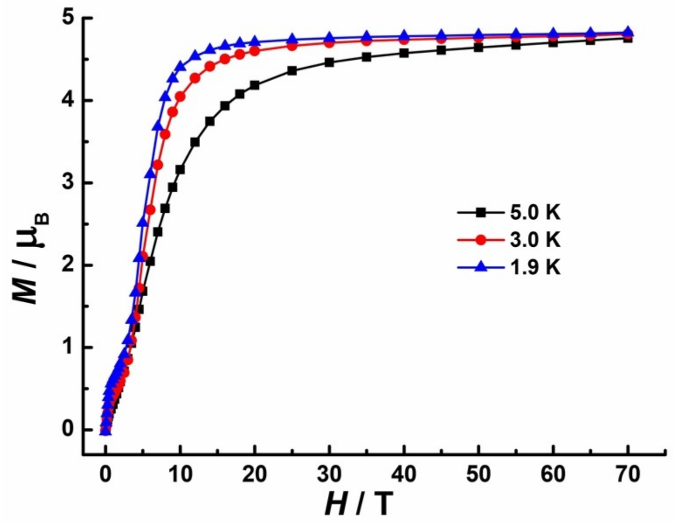

Fig. S6 Molar magnetization (*M*) *vs.* magnetic field (*H*) for **2** at 1.9, 3.0, and 5.0 K.

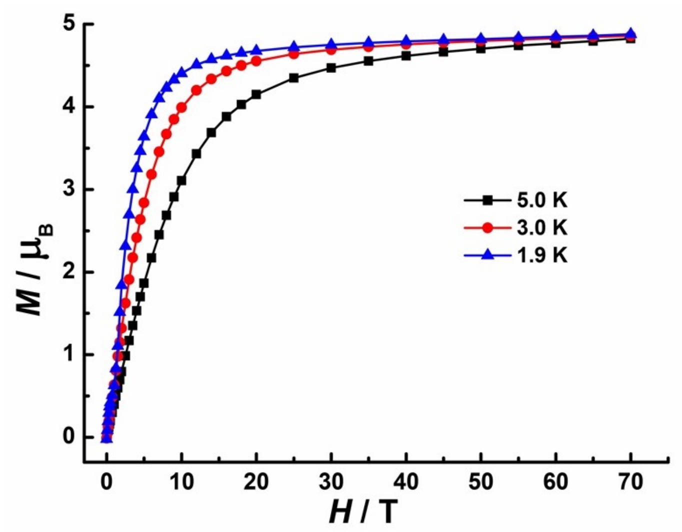

Fig. S7 Molar magnetization (*M*) *vs.* magnetic field (*H*) for **3** at 1.9, 3.0, and 5.0 K.

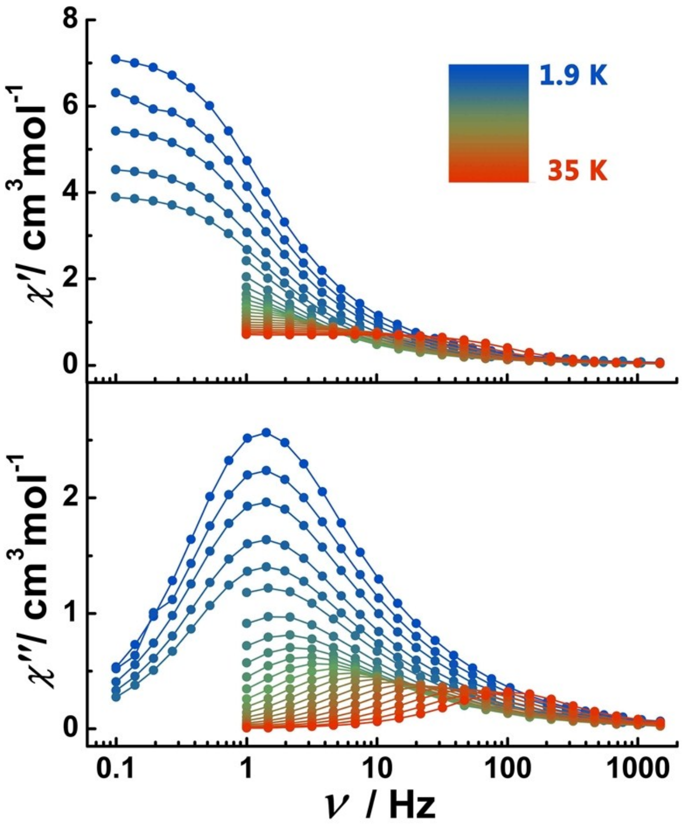

Fig. S8 Temperature and frequency dependent ac susceptibility for **1**.

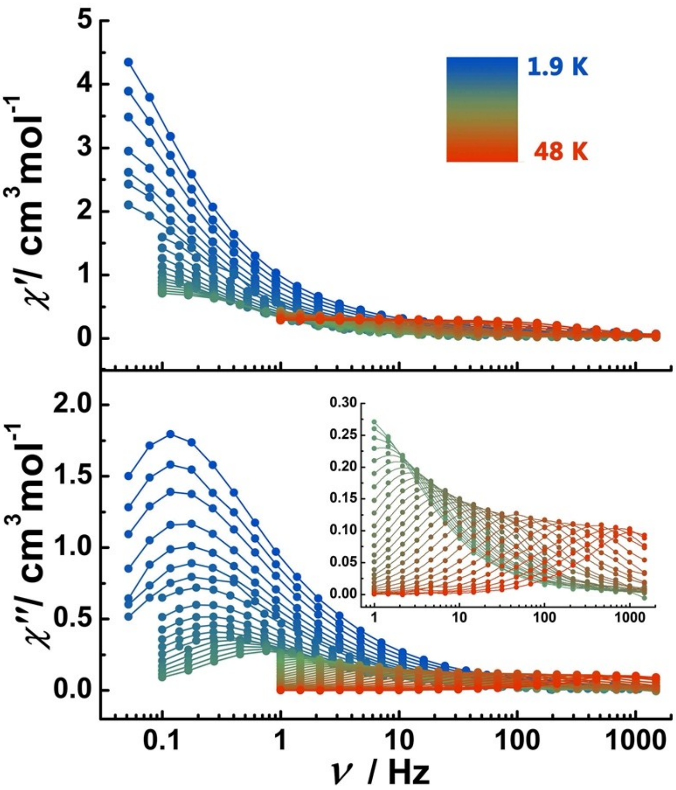

Fig. S9 Temperature and frequency dependent ac susceptibility for **2**. Inset: zoomed in plots of out-of-phase χ'' in the frequency range from 1 to 1488 Hz.

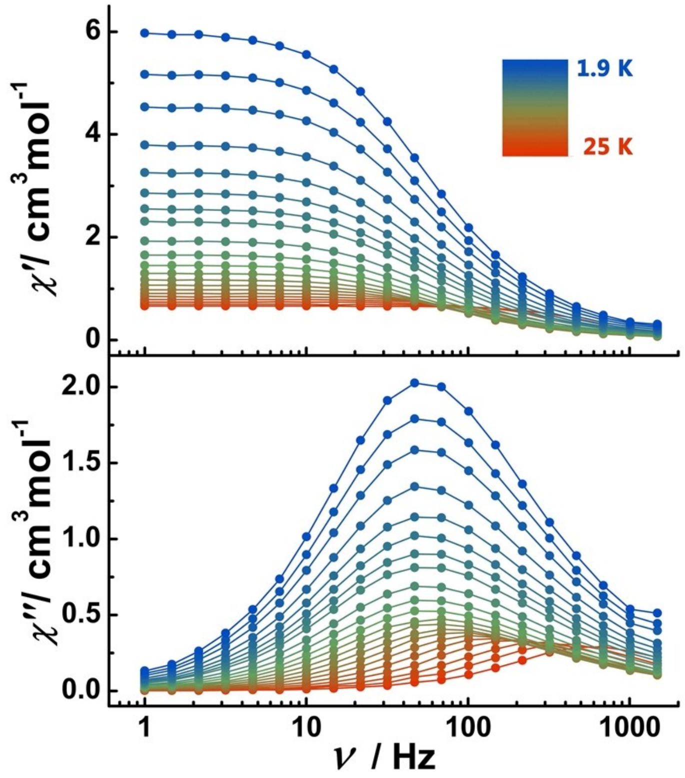

Fig. S10 Temperature and frequency dependent ac susceptibility for **3**.

Arrhenius Plot Fitting.

The temperature and frequency dependence of the magnetic relaxation times for **1-3** were analyzed in terms of multiple relaxation processes, thus many fits were tried to characterize the relaxation pathways. The typical magnetic relaxation pathways are accounted for in equation (1), which is widely used in the literature.[2]

$$\frac{1}{\tau_{obs}} = \frac{1}{\tau_{QTM}} + AH^2T + CT^n + \tau_0^{-1}\exp\left(\frac{-U_{eff}}{k_B T}\right) \qquad (1)$$

Here, $1/\tau_{QTM}$ represents the temperature independent quantum tunneling pathway, AH^2T, the direct relaxation process ($\propto T$), CT^n, the raman relaxation process ($\propto T^n$, n = 4, 5, 7, or 9 typically),[2] and the last term (i.e. the one $\propto \exp(U_{eff}/k_BT)$), the Orbach process. The effective energy barriers were fixed at the value which is obtained from the Arrhenius fitting. The fitting results were listed in Table S5.

Table S4. Parameters obtained from the fitting of the temperature-dependent ac susceptibility for **1**−**3** and corresponding diluted sample for **2**.

Complex	U_{eff} / K	τ_0 / s	τ_{QTM} / s	AH^2	C	n
**1**	221	$1.2(2)\times10^{-7}$	$9.74(2)\times10^{-2}$	-	1.1×10^{-3}	4.3
**2**$_{(pure)}$	615	$1.19(2)\times10^{-11}$	2.1(8)	$1.87(2)\times10^{-2}$	$1.19(10)\times10^{-6}$	5.3
**2**$_{(67\%)}$	668	$5.0(2)\times10^{-11}$	5.85(4)	$8.79(4)\times10^{-2}$	$5.6(4)\times10^{-7}$	5.4
**2**$_{(7.6\%)}$	696	$8.17(5)\times10^{-12}$	$9.09(3)\times10^{5}$	$2.42(3)\times10^{-5}$	$3.64(5)\times10^{-6}$	4.8
**3**	120	$5.22(4)\times10^{-7}$	$2.68(6)\times10^{-3}$	0.499(2)	0.0749	3

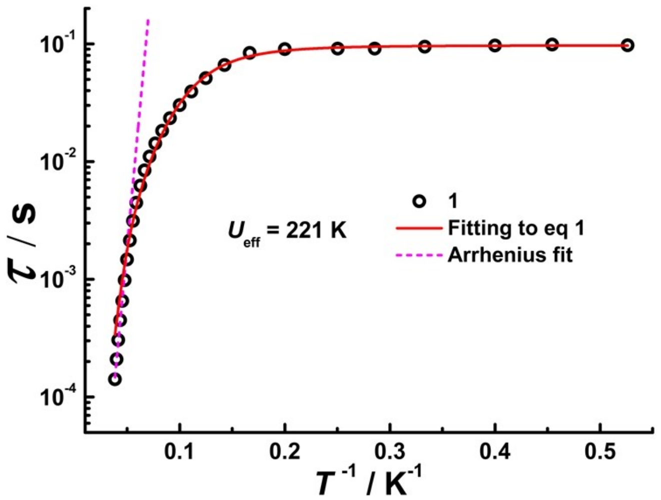

Fig. S11 Plot of τ versus T^{-1} for **1**, obtained under zero-field over the temperature range 1.9–26 K. The red line represents the fitting to eq 1, pink represents the Arrhenius fitted result.

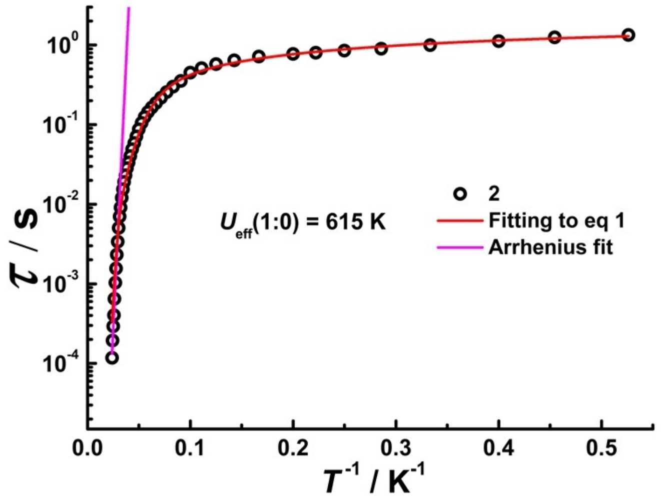

Fig. S12 Plot of τ versus T^{-1} for **2**, obtained under zero-field over the temperature range 1.9–42 K. The red line represents the fitting to eq 1, pink represents the Arrhenius fitted result.

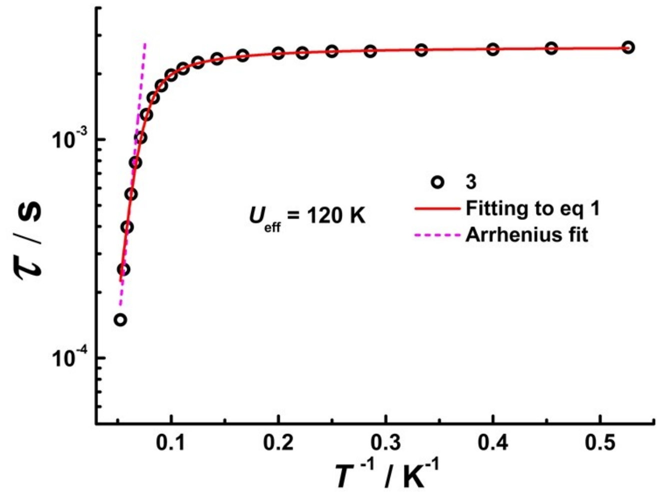

Fig. S13 Plot of τ versus T^{-1} for **3**, obtained under zero-field over the temperature range 1.9–19 K. The red line represents the fitting to eq 1, pink represents the Arrhenius fitted result.

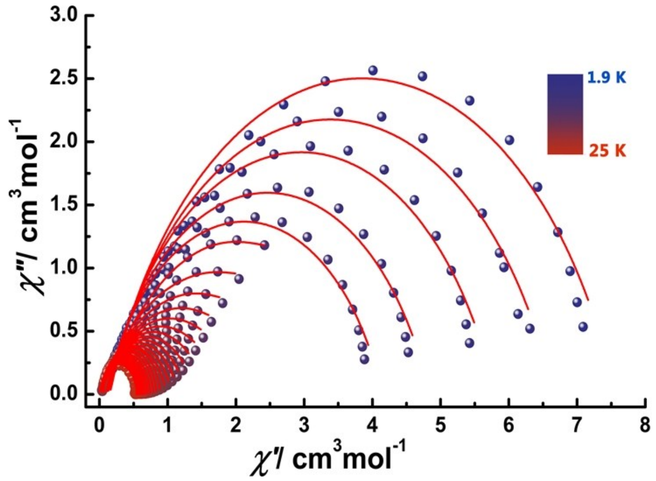

Fig. S14 Cole-Cole plots for **1** at zero-field between 1.9 and 22 K. The solid lines indicate the best fits to the experiments with the generalized Debye model.

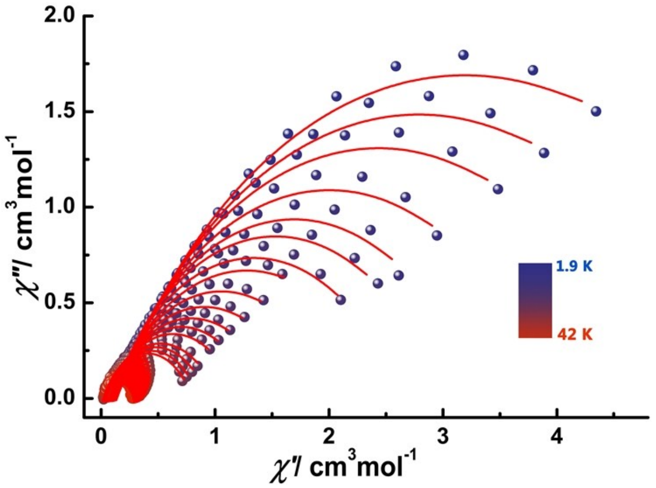

Fig. S15 Cole-Cole plots for **2** at zero-field between 1.9 and 42 K. The solid lines indicate the best fits to the experiments with the generalized Debye model.

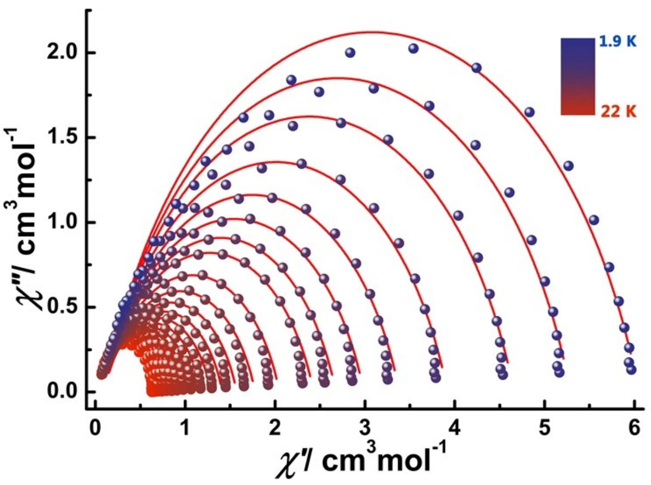

Fig. S16 Cole-Cole plots for **3** at zero-field between 1.9 and 25 K. The solid lines indicate the best fits to the experiments with the generalized Debye model.

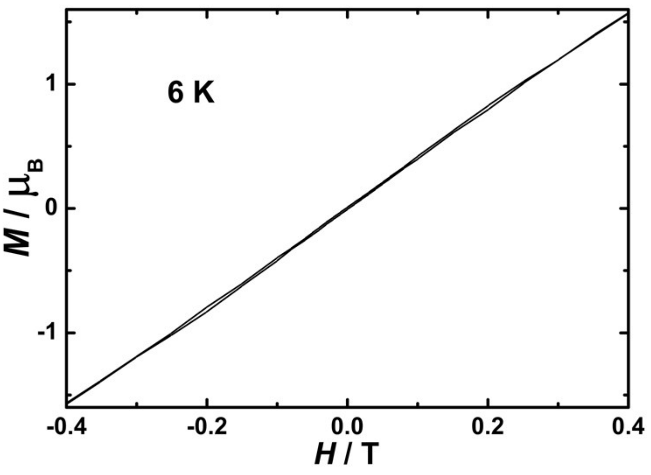

Fig. S17 Zoomed in magnetic hysteresis of **1** at 6 K. Opening is observed at $H \neq 0$.

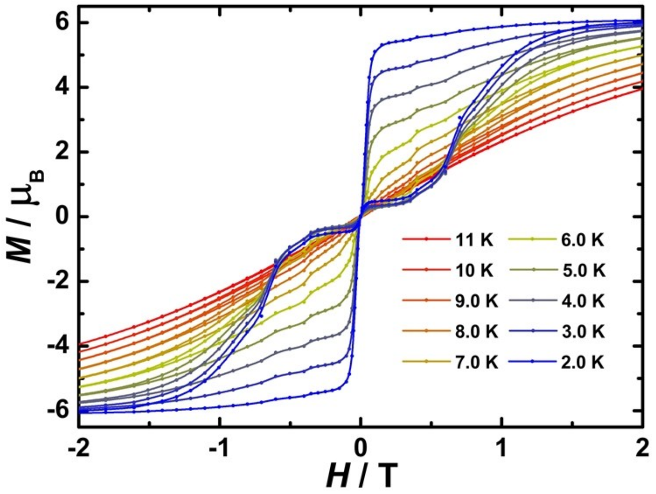

Fig. S18 The magnetic hysteresis of diluted sample of **2** (Dy:Y = 2:1) at indicated temperatures. Solid lines are guides for the eye.

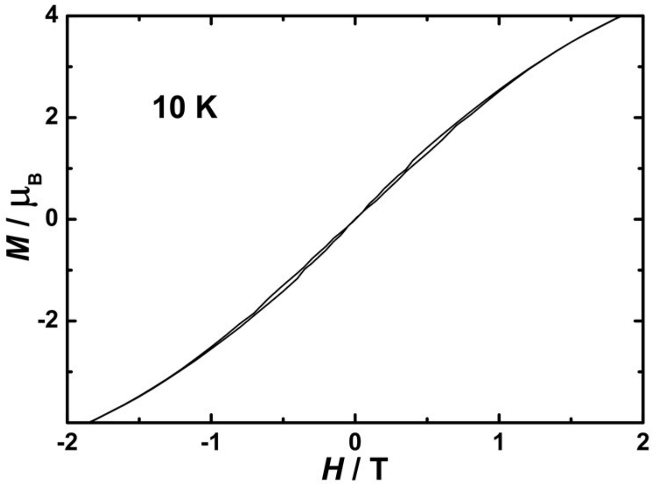

Fig. S19 Zoomed in magnetic hysteresis of diluted sample of **2** (Dy:Y = 2:1) at 10 K. Opening is observed at $H \neq 0$.

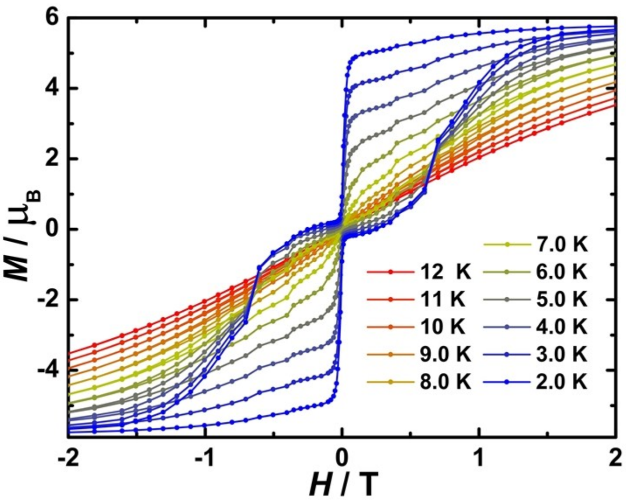

Fig. S20 The magnetic hysteresis of diluted sample of **2** (Dy:Y = 1:12) at indicated temperatures. Openings in the hysteresis are observed up to 11 K at $H \neq 0$.

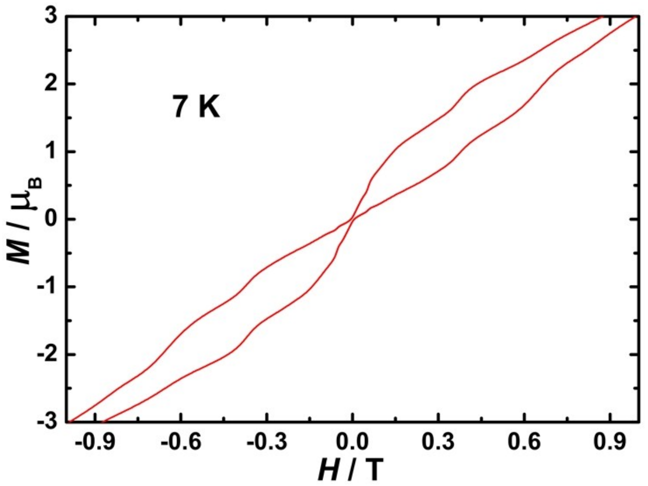

Fig. S21 Zoomed in magnetic hysteresis of diluted sample of **2** (Dy:Y = 1:12) at 7 K. The coercivity is observed up to 7 K at $H = 0$.

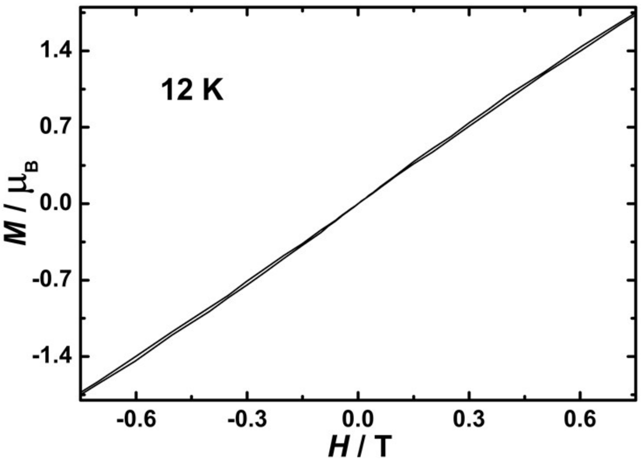

Fig. S22 The magnetic hysteresis of diluted sample of **2** (Dy:Y = 1:39) at 12 K. Opening in the hysteresis is observed up to 12 K at $H \neq 0$.

Table S5. Geometry parameters and energy barriers for β-diketonate dysprosium derivatives.

Complex	d_{pp} / Å	U_{eff} / K	Reference
[Dy(H_2O)(acac)$_3$]	2.4878	64.3	10b
[Dy(phen)(acac)$_3$]	2.6004	64	10e
[Dy(dpq)(acac)$_3$]	2.6208	136	10f
[Dy(dppz)(acac)$_3$]	2.5962	187	10f
[Dy(bpy)(TTA)$_3$]	2.559	58	10d
[Dy(phen)(TTA)$_3$]	2.6219	85	10d
[DyLz$_2$(*o*-vanilin)$_2$]OTf	2.6076	112	This work
[DyLz$_2$(*o*-vanilin)$_2$]Br	2.6386	221.8	This work
[DyLz$_2$(*o*-vanilin)$_2$]NO_3	2.6499	696	This work

Table S6. *Ab initiog*-tensors, energies and deviations angles for the eight Kramer doublets of the $^6H_{15/2}$ ground multiplet of Dy^{III} in **1** (left), **2** (middle) and **3** (right). Deviation angles correspond to the angle between the main magnetic axes of two successive doublet states.

spin-orbite states	Anisotropy tensor (g_X, g_Y, g_Z)	Relative energies (cm^{-1})	Deviation angle (°)	Anisotropy tensor (g_X, g_Y, g_Z)	Relative energies (cm^{-1})	Deviation angle (°)	Anisotropy tensor (g_X, g_Y, g_Z)	Relative energies (cm^{-1})	Deviation angle (°)
**1**	0.00<br>0.00<br>19.72	0	-	0.00<br>0.00<br>19.8	0	-	0.00<br>0.00<br>19.68	0	-
**2**	0.08<br>0.08<br>16.67	202	4	0.00<br>0.00<br>17,00	255	5	0.20<br>0.21<br>16.43	181	3
**3**	0.09<br>0.10<br>13.24	346	8	0.18<br>0.24<br>13.92	471	2	1.28<br>1.32<br>12.75	303	9
**4**	1.83<br>2.29<br>10.15	433	31	3.75<br>5.29<br>10.46	595	85	2.70<br>5.29<br>8.81	379	24
**5**	7.60<br>5.46<br>1.55	485	96	0.42<br>3.77<br>9.27	646	90	3.73<br>4.09<br>11.57	428	69
**6**	2.47<br>3.47<br>11.13	524	27	1.10<br>3.21<br>10.83	721	25	1.04<br>1.06<br>17.63	485	8
**7**	0.79<br>2.41<br>16.4	556	31	0.47<br>1.82<br>15.56	741	30	0.16<br>0.51<br>19.39	524	90
**8**	0.04<br>0.05<br>19.54	615	66	0.07<br>0.41<br>19.35	775	55	0.02<br>0.11<br>19.71	560	90

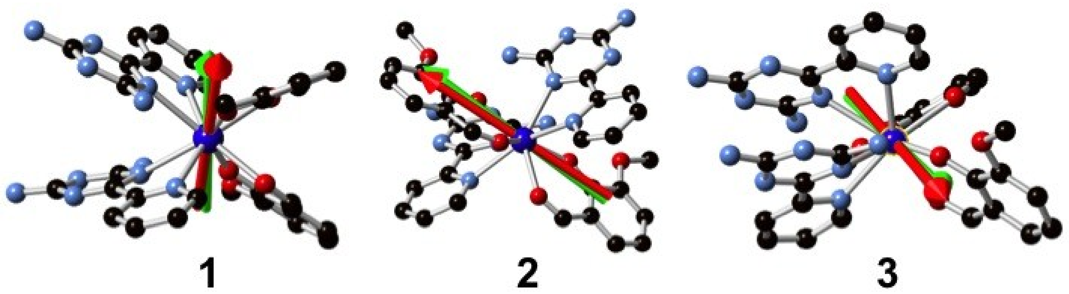

Fig. S23 Axes orientations of the ground and first excited states in **1** (left), **2** (middle) and **3** (right).

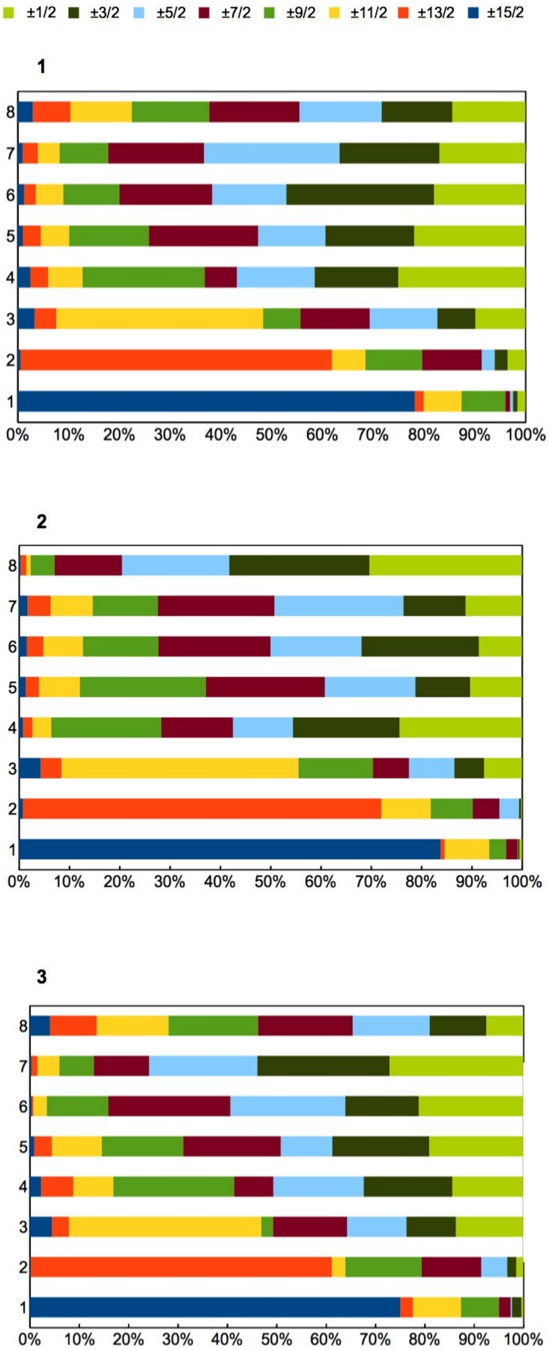

Fig. S24 *Ab initio* wave-functions of the eight Kramer doublets of the $^6H_{15/2}$ ground multiplet of Dy^{III} in **1-3**, in term of M_J eigenstates. The vertical axis stands for the states and the horizontal axis for the percentage of each M_J eigenstates.

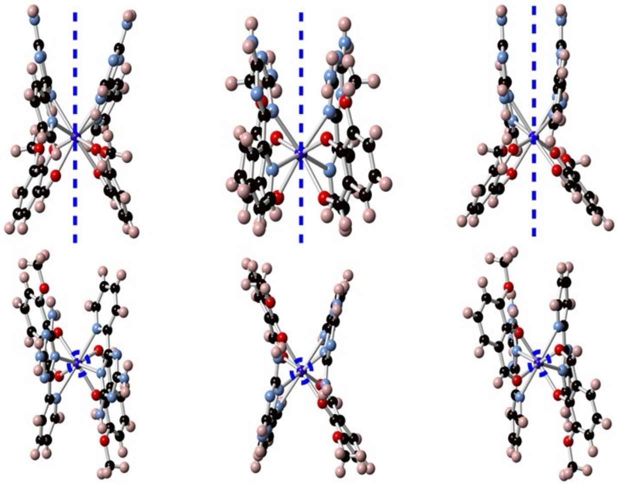

Fig. S25 Location of the pseudo-C_2 molecular axis (dashed blue lines) in **1** (left), **2** (middle) and **3** (right)**.**

References

1. (a) D. Casanova, P. Alemany, J. M. Bofill and S. Alvarez, *Chem. Eur. J.*, 2003, **9**, 1281-1295; (b) S. Alvarez and M. Llunell, *J. Chem. Soc., Dalton Trans.*, 2000, 3288-3303.
2. (a) J. M., Zadrozny, Mihail Atanasov, Aimee M. Bryan, Chun-Yi Lin, Brian D. Rekken, Philip P. Power, Frank Neese and J. R. Long, *Chem. Sci.*, 2013, **4**, 125-138 ; (b) S. Demir, J. M. Zadrozny and J. R. Long, *Chem. Eur. J.*, 2014, **20**, 9524-9529; (c) K. R. Meihaus, S. G. Minasian, W. W. Lukens, S. A. Kozimor, D. K. Shuh, T. Tyliszczak and J. R. Long, *J. Am. Chem. Soc.*, 2014, **136** 6056-6068.
